# Sex and Gender Influences on the Impacts of Disasters: A Rapid Review of Evidence

**DOI:** 10.3390/ijerph22091417

**Published:** 2025-09-11

**Authors:** Carol Muñoz-Nieves, Lorraine Greaves, Ella Huber, Andreea C. Brabete, Lindsay Wolfson, Nancy Poole

**Affiliations:** 1 Centre of Excellence for Women’s Health, Vancouver, BC V6H 3N1, Canada; lgreaves@cw.bc.ca (L.G.); ellahuber95@gmail.com (E.H.); andreea.c.brabete@gmail.com (A.C.B.); lindsay.wolfson@gmail.com (L.W.); npoole@cw.bc.ca (N.P.); 2 School of Population and Public Health, Faculty of Medicine, University of British Columbia, Vancouver, BC V6T 1Z4, Canada

**Keywords:** sex, gender, emergency management, disaster response, public health, climate events

## Abstract

Both sex-related factors and gender-related factors affect the immediate and long term mental and physical health impacts of disasters, including those resulting from public health emergencies, climate-related events, and naturally occurring phenomena. These include sex-specific biological, physiological and genetic processes, mechanisms underlying reproduction, disease outcomes, and stress, immune, and trauma responses. Gendered factors such as roles, relations, identity, and institutional policies that have an impact on caregiving, occupation, gender-based violence, and access to healthcare, also influence the impacts of disasters and emergencies. Sex/gender factors interact with a range of social determinants to affect the equitability of impacts. A rapid review was conducted to examine evidence from Australia, Canada, countries from the European Union, New Zealand, the United Kingdom (UK), and the United States of America (USA) on the influence of sex- and gender-related factors in the context of disasters, such as COVID-19, earthquakes, floods, hurricanes, and wildfires. This article describes and categorizes this evidence with attention to real-world impacts of the interactions between sex, gender, and other equity related factors. Broad considerations for improving research and practices to support more sex and gender research in this area and ultimately, to improve emergency and disaster management, are discussed.

## 1. Introduction

Emergencies and disasters, particularly those related to climate change, are increasing in frequency and intensity. The impacts of emergencies are often unevenly experienced, in that existing inequities are often reinforced or enhanced. Both (biological) sex-related factors as well as (sociocultural) gender related factors affect the impacts of emergencies on people.

Gender issues and inequities, along with those related to income, age, ability, education, race/ethnicity and sexual orientation, are key factors in determining the impacts of emergencies. For example, across the globe, women and children experience greater impacts of disasters than men [1]; women are at greater risk in disasters than men and, in developing countries, disaster-related mortality rates are higher for women than for men [1,2]. Women are more likely to experience PTSD and other mental health issues in response to disasters [3,4], while men may be more vulnerable to suicidality [5], although notable exceptions are reported [6,7]. People who are of low socioeconomic status experience slower recovery post-disaster [8,9]. And those who are socially vulnerable, marginalized, or less able to respond, such as those with physical or mental disabilities [10,11], or who are elderly [10,12], face unique and increased challenges in emergency situations. Emerging research on gender and sexually diverse populations show amplified vulnerabilities linked to discrimination and marginalization [5,13]. In addition, the rates of gender-based violence (GBV) are increased during emergencies [5,10,14] and food [15] and housing insecurity [16,17] often escalated.

Sex-related factors, concerned with biological, physiological, and genetic processes in males and females, also influence impacts of emergencies and disasters. For example, physiological changes during pregnancy, such as reduced lung and functional residual capacity, increased cardiac output, and immune compromise, can increase susceptibility to environmental hazards such as smoke, heat, and infectious agents, which are commonly encountered during disasters [18]. Reduced mobility in late pregnancy may impair a woman’s ability to evacuate or respond quickly in emergency situations [18]. A meta-analytic review found that disaster-related prenatal maternal stress (PNMS) significantly affected multiple spheres of child development. Higher PNMS levels were associated with longer gestational age, larger newborns, higher body mass index (BMI) and adiposity levels, and worse cognitive, motor, socio-emotional, and behavioural outcomes [19].

Despite these differential impacts, emergency management is not always tailored to predict or ameliorate such unequal impacts. In 2023, the Chief Public Health Officer of Canada issued a report indicating that both research and practice could change to decrease inequities and increase resilience among communities in and after emergencies in Canada [20]. If both were improved, advance planning could more often account for sex/gender and equity issues.

This review underpins a larger project aimed at improving emergency response systems in Canada by incorporating sex, gender equity considerations, and a SGBA+ approach into disaster and public health emergency management into a guiding framework, described elsewhere [21]. But improving emergency responses to include and consider relevant sex and gender related factors, and their interplay with a range of factors that contribute to inequities in outcomes, relies in part, on available evidence.

This review is a starting point for assessing the available literature from selected countries for sex/gender/equity factors, categorizing the literature across various sex and gender related categories, and analyzing such evidence using SGBA+. This review therefore provides a detailed basis for future research and identification of impacts, areas, issues, and population groups that require attention to support more sex- and gender-responsive emergency and disaster management. This detailed categorization is supported by examples of research, that go well beyond simple operationalizations or interpretations of sex and/or gender concepts, to illustrate the wide array of impacts that need further research.

## 2. Materials and Methods

We carried out a rapid review of the evidence from selected countries on sex, gender, and equity factors, and the impacts of emergencies and emergency responses, to effectively describe the literature in this area, and ultimately to inform the development of a framework for emergency management in the Canadian context [21].

For this review, we took guidance from the scoping review of rapid review methods by Tricco et al. (2015) [22]. The objective of this rapid review was to identify, examine, and summarize the sex- and gender-related factors that impact emergencies and emergency interventions focusing on the following research question: What do we know about sex, gender, ethnicity, age, socioeconomic status, and/or disability and impacts of emergencies and emergency interventions?

### 2.1. Search Strategy

The search was conducted between 1 August 2024 and 8 August 2024. Four databases were searched, including Medline, CINAHL, PsychInfo, and Cochrane, to identify relevant studies using keywords related to gender, sex, femininity, masculinity, income, and ethnicity, with terms related to emergencies such as natural disasters, avalanche, drought, and hurricane, in the title. This search strategy was designed to find the most relevant studies, streamline the process, and increase specificity to match our research question while minimizing irrelevant results. The search strategy was designed by one researcher (A.C.B), discussed with the entire team, and then the searches were conducted by the same researcher. Search results from all databases were imported into EndNote 20 for deduplication and then uploaded to Covidence for screening.

### 2.2. Literature Screening, Study Selection, and Categorization

**Figure 1:**
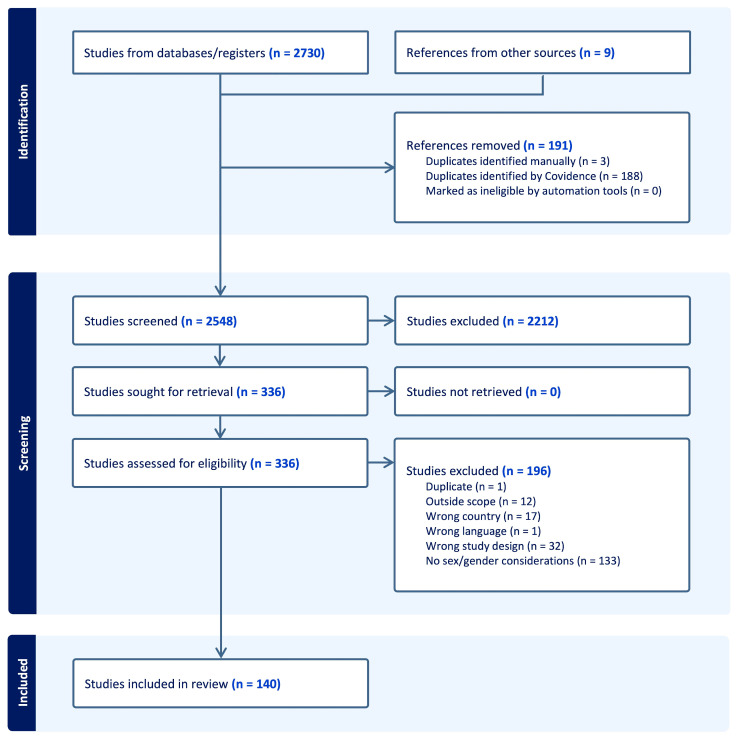
PRISMA chart for this rapid review.

The search across the four databases yielded a total of 2730 results. After removing duplicates, 2548 unique articles remained. Nine papers were added manually. Figure 1 presents the preferred reporting items for systematic reviews and meta-analysis (PRISMA) chart [23], displaying an overview of the literature search process and detailing the total number of articles included and excluded at each screening stage.

In the title and abstract screening phase, five authors (L.G., E.H., A.C.B, L.W., N.P.) calibrated 1% of the unique returns (*n* = 25) according to the screening criteria established a priori. The discrepancies were solved through discussion among all reviewers. The records were title and abstract screened separately by five independent authors (C.M.-N., E.H., A.C.B, L.W., N.P.) in Covidence.

After completing the title and abstract screening, the full texts of the included articles were retrieved, and the full-text screening was calibrated on 3% of the papers (*n* = 10 papers) among all authors. Four authors (C.M.-N., E.H., A.C.B., L.W.) independently screened full text articles in Covidence based on the eligibility criteria. We included English language articles published between 2016 and August 2024 from Australia, Canada, countries from the European Union, New Zealand, the United Kingdom (UK), and the United States of America (USA). In accordance with the terminology used in the Chief Public Health Officer of Canada’s Report on the State of Public Health in Canada 2023, *Creating the Conditions for Resilient Communities: A Public Health Approach to Emergencies* [20], our rapid review focused on the following emergency types: meteorological and hydrological (e.g., avalanche, cold, drought, flood, heat event, hurricane, storm surge, tornado, wildfire), biological (e.g., infectious and communicable diseases, food-borne illnesses, vector-borne diseases, water borne illnesses, zoonotic diseases), and geological (e.g., earthquake, landslide, tsunami, volcano). We excluded studies that focused on conflicts (e.g., mass shootings, arson, civil incident, hijacking, terrorist and cyber-attacks) or technological emergencies (e.g., fire, explosion, hazardous chemicals, transportation accident, infrastructure failure, space event). Following the screening process, included papers were first synthesized by one author (C.M.-N.) and then all authors contributed to reviewing and interpreting the findings.

We included studies that focused on: sex-related (biological) and gender-related (social) factors; sex/gender differences; and sex/gender and intersecting factors according to the categories identified by Greaves and Ritz (2022) [24]. Examining ‘differences’ in males or females, or between, men, women, and gender diverse people, when measuring impacts of emergencies is useful in signalling sex/gender specific issues and identifying areas of further study or inequity. However, examination of differences does not always address or uncover underlying mechanisms for sex and gender related impacts. Investigating the interactions of sex and gender related factors as well as more complex intersecting patterns of influence by race/ethnicity, sexual and gender minority status, ability, class, and age, are more useful approaches to understanding the real-life impacts of emergencies.

## 3. Results

A total of 140 papers were included in the final review. Emergency types included earthquakes, hurricanes, floods, landslides, wildfires, extreme weather and temperatures, and the COVID-19 global public health emergency.

### 3.1. Sex-Related Factors Influencing the Impacts of Emergencies and Disasters

**Figure 2:**
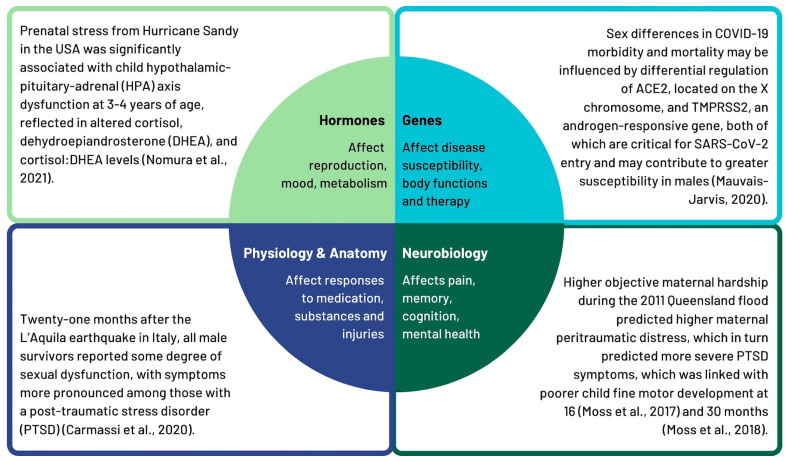
Examples of sex-related factors influencing impacts of emergencies and disasters. (References: Nomura et al. [25]; Carmassi et al. [26], Mauvais-Jarvis [27], Moss et al. [28], and Moss et al. [29]).

We categorized the evidence on sex-related factors (see Figure 2), with examples addressing the differences, instances, and mechanisms reflecting hormones, neurobiology, anatomy, physiology, and genetic evidence. These factors reflect the impacts on female and male bodies. In the evidence reviewed, most of the sex-related impacts were evident in processes of reproduction including pregnancy, fetal health impacts, mental health outcomes, and parenting. 

#### 3.1.1. Hormones

Cohort studies involving pregnant women and their offspring reported associations between disaster-related maternal stress and changes in offspring hormonal regulation and stress reactivity. A study of pregnant and recently postpartum women exposed to the 1998 ice storm in Quebec, Canada found that greater maternal hardship during the storm was linked to higher cortisol levels in their 13-year-old adolescent children when stressed. This suggests that increased maternal stress is associated with more pronounced stress responses in offspring [30]. Females whose mothers reported higher levels of objective distress were found to express higher pre-stressor cortisol levels compared to males [30]. A USA cohort study found that prenatal stress from Hurricane Sandy was significantly associated with child hypothalamic–pituitary–adrenal (HPA) axis dysfunction at 3–4 years of age, reflected in altered cortisol, dehydroepiandrosterone (DHEA), and cortisol: DHEA levels, as well as with increased child anxiety and aggression [25].

#### 3.1.2. Genes

Cohort studies on pregnant women and their offspring also identified associations between disaster-related maternal stress and fetal and child development mediated by alterations in gene expression, epigenetic regulation, and gene–environment interactions. A USA cohort study found that prenatal stress-induced changes in children’s stress hormone levels due to Hurricane Sandy were accompanied by significant reorganization of the placental transcriptome via vascular, immune, and endocrine gene pathways. Researchers found that many of the most altered genes were uniquely expressed in syncytiotrophoblast (STB) cells and contained glucocorticoid response elements in their promoter regions, with several vascular and immune-related gene sets mediating the relationship between prenatal stress and childhood outcomes [25].

A cohort study on the 2011 Queensland flood in Australia found sex-specific patterns in how children’s genotypes moderated the impact of disaster-related prenatal maternal stress on Autism Spectrum Disorder (ASD) traits at 30 months [31]. In males, higher ASD traits were linked to a specific gene (5-HTTLPR LL) combined with disaster-related prenatal maternal stress, while in females, higher ASD traits were connected to a different gene variation (5-HTTLPR LS or SS) combined with disaster-related prenatal maternal stress [31].

A cohort study on the 1998 Quebec ice storm found sex-specific genetic influences on the impact of prenatal maternal stress on children’s hippocampal development at age 13 [32]. In females, objective maternal hardship was linked to right hippocampal volume. Brain Derived Neurotrophic Factor (BDNF) and catechol-O-methyltransferase (COMT) genotypes were associated with left hippocampal volume in both sexes. Moreover, single nucleotide polymorphisms in the COMT genotype moderated the effects of maternal objective distress in males and subjective distress in females on right hippocampal volume [32]. Meanwhile, negative maternal cognitive appraisal of the ice storm predicted both lower BMI and central adiposity in 13-year-old children via DNA methylation of diabetes-related genes, suggesting a protective role of epigenetics [33]. Finally, a cohort study in Puerto Rico found that stage of gestation at the time of Hurricane Maria was associated with significant differences in DNA methylation in infants, mostly for those who were at 20–25 weeks of gestation when the hurricane struck [34].

A review paper on the COVID-19 pandemic explored why inflammatory immune responses to SARS-CoV-2 were more elevated in men and associated with more lethal outcomes than in women. Based on available studies, reviewers suggested that this may be influenced by differential regulation of ACE2, located on the X chromosome, and TMPRSS2, an androgen-responsive gene, both of which are critical for SARS-CoV-2 entry and may contribute to greater susceptibility in males. Additional studies are needed to confirm these interpretations [27]. Male sex also appeared as a COVID-19 disease severity determinant in a study of 257 inpatients across 13 Spanish hospitals [35].

#### 3.1.3. Neurobiology

Several studies found associations between disaster exposure and maternal mental health morbidities among pregnant women. A USA study found that the frequency of PTSD and depression was higher in pregnant women with high exposure to Hurricane Katrina compared to women without high hurricane exposure [36]. The risk of PTSD and depression increased with an increasing number of severe experiences of the hurricane [36].

Cohort studies of pregnant populations and their offspring identified associations between disaster-related maternal stress and fetal and child brain structure, functional connectivity, and neurodevelopment. Sex-specific effects of prenatal maternal stress from the 1998 Quebec ice storm were observed on children’s amygdala development and externalizing symptoms at age 11.5 [37]. Prenatal maternal stress from the 1998 Quebec ice storm also resulted in enlarged brain regions and increased functional connectivity in offspring at age 19, including areas such as the thalamus, hippocampus, and occipital lobe, with the thickness of specific regions, such as the left occipital pole, linked to the timing of maternal distress [38]. This cohort study also showed that prenatal maternal stress was linked to weaker brain connections between emotional and sensory areas at age 19, which was associated with more difficulty in practical language [39]. Greater emotional stress in mothers was connected to weaker brain connections between emotional and motor areas, leading to a more distant personality in their children [39].

A cohort study on the 2011 Queensland flood found that maternal objective hardship was linked to sex-specific changes in hair trace element levels in children, with corresponding changes mediating suboptimal behavioural outcomes at age four [40]. Higher objective maternal hardship predicted higher maternal peritraumatic distress, which in turn predicted more severe PTSD symptoms, which was linked with poorer child fine motor development at 16 [28] and 30 months [29]. More severe objective hardship during pregnancy from the 2011 Queensland flood in Australia was linked to higher sleep problem scores in children at 2.5 years [41]. Some contrasting outcomes were also reported. Higher levels of prenatal maternal stress were positively related to infant motor development at 2 months, yet at 6 and 16 months of age there was a negative association, particularly if flood exposure occurred later in pregnancy and if mothers had negative cognitive appraisals of the event [42]. In contrast, negative maternal cognitive appraisal of the flood predicted lower attention problem scores at the same age [41].

#### 3.1.4. Physiology and Anatomy

Studies that observed somatic symptoms and disruptions in biological rhythms following disaster exposure found sex-specific variations in sleep, appetite, weight regulation, sexual functioning, and other physical symptoms. For example, 21 months after the L’Aquila earthquake in Italy, high school females reported significantly higher rates of somatic symptoms such as headaches, gastrointestinal issues, and altered sensitivity to pain or temperature stimuli, compared to males [43]. Further studies reporting on the L’Aquila earthquake found that females experienced more frequent disruptions in sleep, weight, and appetite, while males experienced sexual dysfunctions [44]. Sexual dysfunction was more pronounced in males with PTSD, although all males affected by the disaster experienced some level of sexual dysfunction symptoms [26]. An increase in sexual dysfunctions were also observed in a female-only Polish study during the COVID-19 pandemic [45]. A USA study also reported sleep disturbances in children and adolescents exposed to Hurricane Harvey. Females showed longer total sleep time, greater sleep efficiency, and less wake time after sleep onset compared to males [46].

Review studies reported associations between maternal disaster exposure and adverse birth outcomes, especially in relation to wildfire exposure. According to an integrative review, maternal wildfire exposure in high income countries was associated with the following: increased rates of gestational diabetes mellitus and gestational hypertension; increased mental health morbidity; impacts on birth weight and length of gestation; differences in the secondary sex ratio; higher incidence of birth defects; and reduction in breastfeeding among evacuated women [47]. In a global systematic review and meta-analysis, wildfire smoke exposure was associated with preterm birth, low birth weight, and small for gestational age infants [48]. Another systematic review found exposure to ozone, fine particulate matter, or high temperatures to be associated with a higher risk of premature childbirth and below-normal birth weight in USA studies [49]. A global systematic review and meta-analysis found that maternal exposure to hurricanes was associated with a higher risk of pre-term birth and lower birth weight [50]. A review of studies on nine earthquakes and four other disasters found a decline in the male-to-female ratio of newborns in various locations worldwide, with the exception of after Hurricane Katrina, which showed an increase in the birth sex ratio. The decreased sex ratio was described as being potentially attributed to immunological causes [51].

In short, a range of studies on disasters, pandemics, and emergencies have shown differential impacts on female and male bodies and have identified some underlying processes such as reproduction and mechanisms such as stress, trauma, genetic, and immune responses as key explanatory factors.

### 3.2. Gender-Related Factors Influencing the Impacts of Emergencies and Disasters

**Figure 3:**
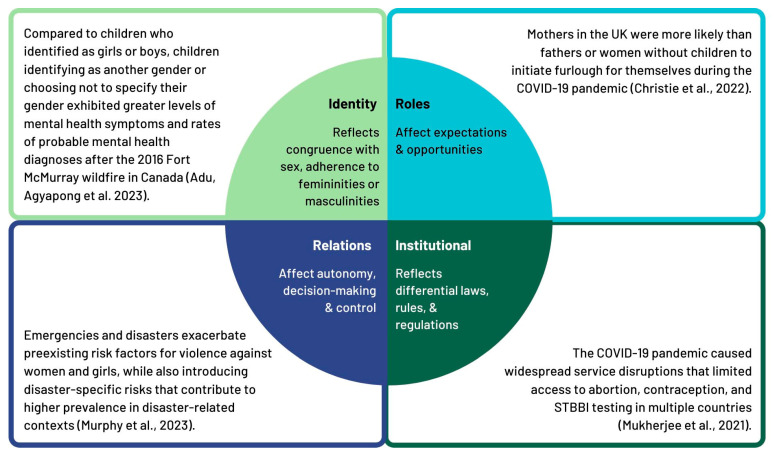
Examples of gender-related factors influencing impacts of emergencies and disasters. (References: Adu et al. [52], Murphy et al. [53], Christie et al. [54], Mukherjee et al. [55]).

Gender is a multi-faceted concept including gender identity, roles, relations, and institutional practices [24]. These factors have an impact on how men, women, and gender diverse people experience emergencies, as well as the responses to such emergencies. In Figure 3, we categorized the evidence on gender-related factors according to these categories and provided examples.

#### 3.2.1. Identity

Gender identity reflects a range of features of a person’s perception of oneself, congruence between sex and felt gender, and their degree of adherence to masculinities or femininities. Such perceptions of femininity, masculinity, and one’s gender identity can influence perceived need for care [56], risk perception [57], and risk-taking behaviours during emergencies [57].

For example, women were more likely than men to receive information, medication and psychological help one year after the 2016 Fort McMurray wildfire in Canada [56]. In a nationwide analysis of flood- and landslide-related deaths in Italy (1965–2014), men were overrepresented in flood-related deaths across most age groups, up to age 89, and in landslide-related deaths up to age 79, while women were overrepresented in flood fatalities above age 70 and landslide-related fatalities between ages 60 and 79 [58]. Despite demographic, cultural, and economic changes over time, these differences remained consistent, suggesting both differing risk-taking behaviours and unequal exposure to geo-hydrological hazards among women and men, which the authors suggested was also connected to gender dynamics in occupational and social roles in Italy [58].

Children identifying as another gender or choosing not to specify their gender exhibited greater levels of mental health symptoms and greater rates of probable mental health diagnoses after the 2016 Fort McMurray wildfire in Canada compared to children who identified as girls or boys [52]. Children who identified as girls had worse mental health scores than boys [52].

#### 3.2.2. Roles

Hegemonic gender roles are heavily influenced by societal norms and often automatically assigned to males and females, determining private and public occupations, social functions, and health outcomes. Such roles can impact how men, women, and gender diverse individuals behave during, and are impacted by, disasters and emergencies. For example, there is evidence that adherence to traditional feminine or masculine gender roles has been linked to variations in mental health outcomes following disasters. A study of Hurricane Harvey survivors in the USA found that individuals with stricter adherence to traditional feminine gender roles exhibited a stronger relationship between trauma severity and both PTSD symptoms and depression. Those with stricter adherence to traditional masculine roles showed a stronger relationship between trauma severity and PTSD symptoms, while survivors with more androgynous gender role orientations demonstrated more adaptive, situation-specific coping skills [59].

Evidence on family and social roles during and after disasters highlighted gender-related dynamics that influenced both behaviours and health outcomes. A study conducted in Lorca, Spain, after the 2011 earthquake found that traditional gender roles were largely reinforced during disaster response efforts. Women reported retrieving household necessities such as clothing and medication to support their families, while men described engaging in tasks deemed ‘risky’, such as gaining access to their damaged homes [60]. During Hurricane Sandy in the United States, most gasoline exposure cases involved men, while most carbon monoxide exposure cases involved women [61], providing evidence on how gendered household roles can have an effect on exposure patterns. Research on the COVID-19 pandemic highlighted how gendered childcare responsibilities can shape employment decisions during and after emergencies. For example, a UK study found that mothers were more likely than fathers or childless women to initiate furlough for themselves during this pandemic [54].

Research on occupational roles during the COVID-19 pandemic showed disproportionate impacts on women in healthcare. In the USA, being female predicted higher levels of depression and burnout among surgical residents [62]. A qualitative study reported that women healthcare providers faced major changes and disruptions in care delivery and work environments, with elevated potential for compassion fatigue and secondary traumatic stress [63].

#### 3.2.3. Relations

Gender relations are often prescribed by societies and cultures, and affect life experiences for men, women, and sexual and gender minority individuals. Such relations often determine issues such as power, decision making, authority in private or public spheres, and behaviours. In emergencies, gender relations may reflect typical sociocultural influences or stereotyping, and gendered assumptions emerge, resulting in differential experiences between gender groups.

For example, a global review of studies revealed that many preexisting risk factors for violence against women and girls (VAWG) were intensified in a range of settings affected by natural disasters and other crises. Poverty and economic stress, men’s substance use, exposure to violence, changing gender roles in contexts of inequitable gender norms, and a lack of social support were some of the risk factors associated with men’s perpetration of, or women’s experience of, violence [53].

The mental health impacts of GBV, including intimate partner violence (IPV), during and after disasters have also been documented. Women in the USA who experienced post-disaster GBV and IPV were significantly more likely to develop PTSD symptoms, major depressive disorder, heightened depression symptoms, and suicidal ideation, according to a review of studies [64]. Protective factors against post-disaster IPV were non-urban settings, a strong sense of community and social cohesion, and integration into the workforce [64].

Gender dynamics in parent–child relationship quality [65] and parent–child recollection patterns [66] following disasters were shown to have an effect on children’s health. Following Hurricane Georges in Puerto Rico, high-quality parent–child relationships were associated with fewer medical issues in boys at 18 months and in girls at 30 months [65]. In contrast, girls whose parents did not feel emotionally close to them experienced more medical problems at 30 months than any other group [65]. In another study, youth anxiety related to recollections of a devastating USA tornado was associated with caregivers’ reinforcement of negative emotional expression, with these patterns generally being stronger in girls than in boys [66], echoing broader patterns in which caregivers were more likely to discuss negative emotions with daughters than sons [66].

Gender relations matter in more positive ways as well in the context of disasters and emergencies. In a qualitative study, pregnant and lactating women affected by the 2009 L’Aquila earthquake in Italy emphasized the importance of reconfigured relationships and the central role of partner and family support for their health and wellbeing [67]. Women also highlighted the need for spaces to share experiences and practices with other mothers [67].

#### 3.2.4. Institutional

Institutional gender manifests in practices, policies, and priorities that reflect gendered approaches and create gendered impacts. For example, funding for sex/gender specific health or social services, tolerance of pay inequity between women and men, or lack of funded childcare have gendered impacts on men, women, and gender diverse people. Such gendered priorities or practices also manifest in emergencies.

For example, research on sexual and reproductive health issues in emergencies found that access to contraception was one of the biggest challenges faced by women during natural disasters in Organisation for Economic Co-operation and Development (OECD) member countries [49]. A global review further found that the COVID-19 pandemic resulted in service disruptions that affected access to abortion, contraceptives, sexually transmitted and blood borne disease (STBBI) testing, and changes in sexual behaviours, menstruation, and pregnancy intentions [55].

Studies also illustrated the impact of institutional and systemic factors on the health and wellbeing of pregnant and breastfeeding populations during disasters. Pregnant women in the US Virgin Islands [68], Puerto Rico [69,70], and Italy [67] experienced health risks related to disrupted food access, unsafe environments, reduced psychosocial support, and limited access to maternity care after earthquakes [67] and hurricanes [68,69,70]. According to a review, challenges specific to breastfeeding during disasters included decreased breastfeeding self-efficacy, lack of knowledge and resources, and over-reliance on formula baby milks [71]. On the other hand, facilitators included privacy, community and family support, adaptation of professional breastfeeding support to the local context, and pre-existing breastfeeding practice [71]. Breastfeeding women also experienced difficulties with formula feeding because of a lack of clean water, supplies, and access to infant formula [71].

Additional research has linked these disruptions to negative mental health outcomes, including postpartum depression. In Puerto Rico, feeling unsafe and struggling to access food had the strongest associations with postpartum depression during and after Hurricanes Maria and Irma in 2017 [70]. In rural Australia, nurses reported that disasters augmented stressors on perinatal women in connection to limited access to support and increased isolation, all of which affect mental health outcomes [72]. During the COVID-19 pandemic, the prevalence of postpartum depression symptoms increased by at least 34% compared to the pre-pandemic period, with risk factors including anxiety about infection, socioeconomic pressures, social isolation, and disrupted prenatal and postnatal care [73].

Studies reported on the impact of evacuation stressors on maternal health and the importance of maternal social support for children’s resilience and wellbeing. For example, evacuation stressors significantly predicted mothers’ post-traumatic stress, anxiety, and depression symptoms three months after Hurricane Irma in the USA [74]. Children whose mothers had adequate social support after the 2013 Calgary flood in Canada reported higher resilience scores than children whose mothers did not have adequate social support [75]. Further, children of mothers over 25 years old reported higher resiliency scores than the children of younger mothers affected by the 2013 Calgary flood [46].

Research on the COVID-19 pandemic showed impacts on parenting stress and child wellbeing due to both preexisting and pandemic-related socioeconomic contexts. In a USA mothers-only study, mothers who experienced employment loss early in the COVID-19 pandemic were more likely to score higher on child abuse risk measures [76]. Meanwhile, mothers whose children had relied on school meals before the pandemic reported greater difficulty feeding their children and increased conflict with them during the pandemic period [76].

#### 3.2.5. Sex and Gender Differences in the Impacts of Emergencies and Disasters

Across the reviewed studies, sex and gender differences were observed in the impacts of disasters and emergencies on a range of outcomes—including post-traumatic stress, anxiety, depression, substance use, and physical health—frequently suggesting heightened vulnerability among females, girls, and women across age groups and disaster types. Exceptions were observed around suicidality and some substance use outcomes, where males, boys, and/or men were found to be more vulnerable.

For example, studies that reported on the prevalence and comparative rates of PTSD following disasters showed higher rates among females, girls, and women compared to males, men, and boys. This was observed in studies of children and adolescents following different disasters: earthquake [77,78,79], flood [77] and hurricane [80] exposure and in a study of wildfire-exposed adults [81]. Evidence also indicated greater PTSD symptom severity in young females following wildfire [82] and earthquake [83] exposure, particularly in re-experiencing and arousal symptoms [82]. Other studies found worse outcomes for females related to PTSD symptom trajectories after disaster exposure [84,85,86], as well as general psychological and mental distress following hurricane exposure [87] and during the COVID-19 pandemic [88].

Other mental health impacts were noted. Several studies found that females experienced higher rates of anxiety [81,89,90,91] and depression [80,81,90,91] following disaster exposure compared to males. This was found in studies of children and adolescents after hurricane exposure [80] and in studies of earthquake- [90] and wildfire-exposed [81,89] adults, as well as adults during the COVID-19 pandemic [91]. However, a review of COVID-19 studies also reported mixed and no sex differences among adults [91]. On suicidality, a USA study from the first year of the COVID-19 pandemic found significantly more suicides among male youth than previously observed figures [88].

Child and adolescent studies found that males and boys more often displayed externalizing behaviours (e.g., aggression, hyperactivity) [83,92,93] while females and girls tended to exhibit more internalizing symptoms (e.g., emotional distress) after disaster exposure [83,93]. For example, two years after the 2012 Modena earthquake in Italy, female children and adolescents reported more emotional problems, while males showed higher levels of hyperactivity, conduct problems, and peer-related issues [83]. Compared to males and boys, females and girls displayed more understanding of their disaster-related emotions [94], belief in others after a disaster [95], resilience [75], and post-traumatic growth [96].

Findings on substance use after disaster exposure suggested complex sex and gender differences, with females, girls, and women at times exhibiting decreased risk for substance use compared to males, men, and boys, while in other cases, were at increased risk for specific types of substance use. For example, a Canadian study found that female sex had a protective effect against risk of drug or alcohol dependency among adults one year after the Fort McMurray wildfire [97]. Among young adults, a higher proportion of females presented with problematic drug use 18 months after the Fort McMurray wildfire, while a higher proportion of males engaged in high risk drinking and experienced moderate to high nicotine dependence [81]. In a USA study, greater family exposure to tornadoes was associated with a greater number of cigarettes smoked among female but not male youth [98]. In a study of traumatic stress and substance use among Puerto Rican youth after Hurricane Maria, girls, who had significantly higher PTSD symptoms scores than boys [99], were also significantly more likely to abstain from substance use [99]. Among girls, substance use was more strongly associated with PTSD-related irritable behaviour and angry outbursts [99]. In boys, substance use had stronger associations with physiological reactivity to trauma reminders [99].

There were also reported sex differences reflecting physical health conditions, hospitalizations, and outcomes. Studies examining respiratory [100,101,102] and cardiovascular [101] outcomes after disaster exposure found that females often faced higher risks of adverse outcomes compared to males, especially among those aged 65 and older [100,101]. A cross-sectional study found that females over 65 had significantly higher risks of respiratory-related hospital admissions due to wildfire smoke compared to males in western USA between 2004 and 2009 [22]. Similarly, a USA case–control study found that females over 65 faced greater risks of cardiovascular and respiratory diseases than elderly males immediately, 4-month, and 12-month after Superstorm Sandy in New York State, compared to the five years prior [23]. In another case–control study, females were more likely than males to be diagnosed with chronic bronchitis in the emergency department during the 2012 heatwave in Douglas County, Nebraska, compared to the same period in 2011 [24]. On functional outcomes, a 12-year USA study on in mid-life and older adults exposed to Hurricane Sandy found that women experienced more functional limitations on average across all trajectory groups, while the greatest impact of disaster exposure was seen in men [103].

### 3.3. Sex/Gender and Intersecting Considerations

Sex, gender, and many other factors and characteristics interact to amplify impacts of and responses to emergencies. For example, race and age have interacted with rural/urban locations to shape the impact of emergencies [104]. Positive associations between drought exposure and mortality were predominantly observed among White females and males across various drought types in both metropolitan and non-metropolitan counties in Douglas County, Nebraska, USA between 1980 and 2014 [103]. Notably, White females aged 45–54 were the only subgroup to demonstrate a statistically significant positive association with all-cause mortality and long-term drought exposure in both metro and non-metro settings, with a slightly stronger effect observed in non-metro areas [104]. In contrast, protective effects were observed in Black males aged 20–24 and White females over 85, primarily in metro counties [104].

Black students and university staff in the USA and UK experienced COVID-19 against the backdrop of racism as a “pandemic within a pandemic” (Laurencin and Walker, 2020 as cited in [105]), including racial (re)traumatization, loneliness, and isolation. Other themes included precarious employment and exploitation [105]. The race–gender element showed to have diverse impacts amongst Black students and staff, with difficulties experienced by Black women in academia related to receiving less support from educational systems [105]. In a USA study of low-income Black mothers who survived Hurricane Katrina, post-traumatic growth was related to increased racial diversity, improved neighbourhoods, and new educational and economic opportunities post-disaster [106].

Gender identity and sexual identity and orientation have been found to interact with emergency type to create mental health outcomes. A qualitative study of USA lesbian, gay, bisexual, and transgender youth reported an increase in distress during the COVID-19 pandemic from being confined at home with unsupportive parents [45]. Discrimination by and within the emergency sector was also reported. An Australian study examining lesbian and bisexual women’s experiences in emergency management found that the sector frequently failed to address their diverse needs, both as service recipients and as paid staff or volunteers, with participants mentioning issues such as exclusionary language and assumptions, discrimination, and the harmful influence of faith-based organizations [107].

Studies of the sexual health of men who have sex with men (MSM) during the COVID-19 pandemic found an increase in casual sexual encounters, a decrease in the frequency of condomless sex, and greater difficulty in accessing condoms during lockdown [45]. In a USA study, MSM and transgender women endorsed monkeypox exposure mitigation strategies such as: limiting the number of sexual partners (40.8%), avoiding bars, clubs, and other parties (33.4%), becoming abstinent or avoiding sexual activity (24.8%), asking their sexual partners if they had monkeypox symptoms (25.0%), and inspecting their sexual partners to see if they had monkeypox symptoms (24.3%) [108]. Rurality was negatively associated with changes in behaviour, whereas being non-White and having oral sex with a non-primary partner in the past six months were positively associated with changing their sexual behaviours [108].

For women, PTSD, depression, and other significant mental health concerns were recurrent issues post-disaster in the USA, however post-disaster mental illness was particularly acute for women who were also minorities, or who experienced poverty, were elderly, or the primary caregivers for their children [64]. Subpopulations, such as incarcerated women, have also been found to experience disaster-related impacts in particular ways. In a study of incarcerated older women (50+) in California, USA, participants described being locked in their cells for 23 h per day or more, often for days, weeks, or even months at a time in an effort to reduce the spread of COVID-19. These lockdowns and the resulting isolation from loved ones both inside and outside of the prison were detrimental to both their physical and mental health [109]. Further barriers were created by the cessation of group programmes and shift to cell-front mental health services [109].

## 4. Discussion

This rapid review identified a wide-ranging body of evidence showing how sex- and gender-related factors influence the effects of emergencies and disasters, instances or patterns of sex differences in impacts of emergencies, and areas where sex/gender interact with other factors such as race, income, sexual or gender minority status, context, or stage of life. We have consolidated this material under specific categories of sex and gender concepts in order to organize and focus the results. Based on these findings, we can identify broad considerations for improving sex and gender science in the area of disasters and emergency management to advance this area and ultimately to support more sex- and gender-responsive emergency and disaster management.

Several sex-related factors linked to female reproduction are central to how emergencies and disasters affect health, highlighting the need for ongoing monitoring and targeted interventions for pregnant and postpartum women, their children and families, and for emergency management sectors to prioritize these groups. Disasters were shown to significantly impact physical [47] and mental [36,50] health during pregnancy with evidence of both short- and long-term effects of prenatal maternal stress on fetal and child health [25,28,29,30,31,32,33,34,37,38,39,40,41,42]. Research on relational and institutional gender-related factors also emphasized the importance of ensuring adequate breastfeeding support [67,71], psychosocial support for pregnant women [68] and mothers [75,110], and food and environmental security for pregnant and postpartum populations [68,69].

Other sex-related factors identified in the research were linked to stress and trauma responses, which were found to affect both males and females in disaster-related contexts, often with more pronounced effects in females [78,79,80,81,82,83,84,85,86]. Building on this, it is important to incorporate universal trauma-informed approaches that acknowledge and anticipate widespread traumatic experiences in disaster-affected communities and the potential for disaster environments to intensify pre-existing trauma-related symptoms [111]. Equally important is the potential inclusion of sex- and gender-sensitive mental health services as core components of emergency response and recovery, along with substance use services, given the links between disaster exposure, trauma, and substance use [98,99].

Research on biological emergencies, such as the COVID-19 pandemic found sex-related factors affecting differences in inflammatory immune responses and morbidity among males and females, with males exhibiting greater susceptibility to SARS-CoV-2 infection and more severe outcomes [35]. At the same time, evidence on gender-related factors revealed that women were disproportionately affected in terms of employment disruptions, increased caregiving and childcare responsibilities [54], and greater exposure through care-related professions [62,63]. These findings indicate the importance of further sex/gender research in future biological emergencies and pandemics to inform sex/gender responsive treatment relevant to successful disaster planning. Medical guidelines, especially for biological emergencies, should account for sex-based biological differences and impacts, while social relief measures and policies must address gendered vulnerabilities related to employment status and caregiving burdens. In addition, emergency preparedness and response should include targeted support for care workers and reinforce the care sector as a critical component of disaster resilience.

On gender roles, research indicates that strict adherence to traditional roles is associated with poorer mental health outcomes [59], and that these roles are more likely to be reinforced than challenged in disaster contexts [60]. This is relevant to the design of emergency communication and public messaging, which should avoid reinforcing gender stereotypes—such as men as protectors and women as caregivers—and instead promote culturally and context-specific roles and adaptive coping strategies within households and communities.

Research on gender relations showed that disasters exacerbate preexisting risk factors for VAWG, while also introducing disaster-specific risks that contribute to higher prevalence [53]. These risks underpin the importance of international guidance on GBV in disasters to emphasize the precautionary principle, which means advocating for early action in the absence of confirmatory evidence, and urging governments and emergency managers to prioritize prevention and response efforts. [112]. Such efforts need to recognize the dynamics of VAWG, including stigma, shame, and gendered power imbalances that can preclude its recognition, particularly in close communities [113]. Consequently, anti-violence services should be categorized as essential, with additional support allocated during disaster situations. Research on institutional gender further revealed that disasters disrupt access to essential sexual and reproductive health services for women—including safe abortion, contraception, and STBBI testing [49,55], underscoring the need to classify and support these services as critical components of emergency preparedness and response.

Children’s health and wellbeing were shown to be influenced by gender-related dynamics in parent–child relationship quality [65] and parent–child recollection patterns [66] following disasters. Studies related to gender identity revealed that an individual’s perception of their own gender identity can shape their perceived need for mental healthcare [56], how they assess risk, and their willingness to take risks during emergencies [57]. It is important that emergency planners consider these patterns to better tailor health support and interventions for specific groups.

Finally, some studies documented the intersections of sex and gender identity with other factors—such as age [64,104], race [104,105,106], sexual orientation [45,107,108], socioeconomic status [64], and geographic location [104]—that shape differential outcomes for individuals and communities in specific disaster contexts. However, these intersections remain underexplored in disaster research. Additional gaps include the limited examination of sex-related factors beyond reproductive health, the underlying biological mechanisms driving these impacts, and the ways sex- and gender-related factors interact to create impacts and responses to emergencies. There is also a need for more research on the ways sex- and gender-related factors affect males and men, and the specific needs of sexual minority and gender-diverse populations.

### Limitations

The rapid review methodology does not typically include detailed quality appraisal, therefore the included studies may be variable in this dimension. Another limitation of this review is the restriction to English-language articles published between 2016 and August 2024, and to studies conducted in Australia, Canada, countries within the European Union, New Zealand, the UK, and the USA. We undertook this approach as part of the rapid review methodology to facilitate a one-year, streamlined project that responded to a national report on equity in emergency management in Canada. However, this may limit the generalizability of findings to other contexts and exclude relevant research from other geographic regions.

SGBA+ was conducted according to the framework established by the review team (and described in previous sections), independent of how sex and gender were conceptualized in the included studies (or whether they were accurately labelled or were defined at all). While this approach allowed for consistency and accuracy across the review and allowed categorizations beyond simplistic conceptual understandings of sex and/or gender, it may introduce interpretive bias and limit the extent to which our analyses align with the original authors’ intended conceptualizations.

## 5. Conclusions

Findings from our rapid review reveal a wide-ranging but nascent area of research applying sex and/or gender concepts to understanding the impacts of disasters and emergencies. We applied categories of sex and gender to organize and illustrate the literature and inspire further research on these issues. Even so, the review highlights the critical need to integrate sex, gender, and equity considerations into research on impacts of disasters and emergencies, and on emergency management. In our review of existing evidence denoting various impacts of both sex and gender related factors affecting the outcomes and experiences of emergencies, the sex-related evidence is predominantly focused on female reproductive processes and the impacts on fetal and child health, as well as related mental health issues. Clearly, sex-related factors could be more broadly investigated across a range of issues and conditions, and in both sexes The gender related evidence was somewhat more diverse, including issues surrounding gender roles, relations, and sexual and gender minority health.

The evidence is uneven and incomplete across types of emergencies, countries, and populations with many gaps remaining, particularly with respect to sex-related impacts and sex/gender interactions, underlying mechanisms of impacts, and clear links to specific phases of disasters and emergency responses. However, the existing evidence clearly indicates that research on impacts of disasters, and emergency management planning ought to consider sex/gender and equity issues, and that more research is needed to support a comprehensive sex- and gender-based informed approach to emergency management. More evolved and nuanced research would better support the United Nation’s Gender Action Plan to Support Implementation of the Sendai Framework for Disaster Risk Reduction 2015–2030 [114] that promotes gender-responsive approaches and emphasizes the vital role of women’s organizations and other gender equality stakeholders in building localized disaster resilience [114]. This would lead to better practices, such as Gender and Disaster Australia’s national guidelines [115], training [116], and practical tools [117]. In order to reduce further inequities in emergency outcomes and within emergency management, research must rapidly evolve to support better practices that consider sex, gender, and a range of intersecting factors in planning and executing responses to emergencies.

## Data Availability

No new data were created.
